# Cell internalization of 7-ketocholesterol-containing nanoemulsion through LDL receptor reduces melanoma growth *in vitro* and *in vivo*: a preliminary report

**DOI:** 10.18632/oncotarget.24389

**Published:** 2018-02-04

**Authors:** Giovani M. Favero, Jessica L. Paz, Andréia H. Otake, Durvanei A. Maria, Elia G. Caldini, Raphael S.S. de Medeiros, Debora F. Deus, Roger Chammas, Raul C. Maranhão, Sergio P. Bydlowski

**Affiliations:** ^1^ Laboratory of Genetics and Molecular Hematology (LIM31), Department of Hematology, Hospital das Clinicas HCFMUSP, Faculdade de Medicina, Universidade de Sao Paulo, Sao Paulo, SP, Brazil; ^2^ Department of General Biology, Universidade Estadual de Ponta Grossa, Ponta Grossa, PR, Brazil; ^3^ Centro de Investigação Translacional em Oncologia (LIM24), Departamento de Radiologia e Oncologia, Hospital das Clinicas HCFMUSP, Faculdade de Medicina, Universidade de Sao Paulo, Sao Paulo, SP, Brazil; ^4^ Instituto do Cancer do Estado de Sao Paulo (ICESP), SP, Brazil; ^5^ Biochemistry and Biophysics Laboratories, Instituto Butantan, Sao Paulo, SP, Brazil; ^6^ Laboratory for Cell Biology, Department of Pathology, Faculdade de Medicina FMUSP, Universidade de Sao Paulo, Sao Paulo, SP, Brazil; ^7^ Laboratory of Metabolism and Lipids, Heart Institute (InCor), Hospital das Clinicas HCFMUSP, Faculdade de Medicina, Universidade de Sao Paulo, Sao Paulo, SP, Brazil; ^8^ Faculdade de Ciencias Farmaceuticas, Universidade de Sao Paulo, Sao Paulo, SP, Brazil

**Keywords:** 7-ketocholesterol, nanoemulsion, melanoma, cell death, LDL receptor

## Abstract

Oxysterols are cholesterol oxygenated derivatives which possess several biological actions. Among oxysterols, 7-ketocholesterol (7KC) is known to induce cell death. Here, we hypothesized that 7KC cytotoxicity could be applied in cancer therapeutics. 7KC was incorporated into a lipid core nanoemulsion. As a cellular model the murine melanoma cell line B16F10 was used. The nanoparticle (7KCLDE) uptake into tumor cells was displaced by increasing amounts of low-density-lipoproteins (LDL) suggesting a LDL-receptor-mediated cell internalization. 7KCLDE was mainly cytostatic, which led to an accumulation of polyploid cells. Nevertheless, a single dose of 7KCLDE killed roughly 10% of melanoma cells. In addition, it was observed dissipation of the transmembrane potential, evidenced with flow cytometry; presence of autophagic vacuoles, visualized and quantified with flow cytometry and acridine orange; and presence of myelin figures, observed with ultrastructural microscopy. 7KCLDE impaired cytokenesis was accompanied by changes in cellular morphology into a fibroblastoid shape which is supported by cytoskeletal rearrangements, as shown by the increased actin polymerization. 7KCLDE was injected into B16 melanoma tumor-bearing mice. 7KCLDE accumulated in the liver and tumor. In melanoma tumor 7KCLDE promoted a >50% size reduction, enlarged the necrotic area, and reduced intratumoral vasculature. 7KCLDE increased the survival rates of animals, without hematologic or liver toxicity. Although more pre-clinical studies should be performed, our preliminary results suggested that 7KCLDE is a promising novel preparation for cancer chemotherapy.

## INTRODUCTION

Oxysterols are 27-carbon products of cholesterol oxidation that can be formed either by auto-oxidation or by specific enzymatic action [1, 2]. They represent a large class of biologically important regulatory molecules involved in sterol and lipid metabolism, cell proliferation, and cell differentiation, as well as in pathophysiological processes, such as atherosclerosis, mutagenesis, and carcinogenesis [3–6].

Oxysterols have potent effects on cell death processes in a number of cell lines [5, 7, 8]. The cytotoxicity attributed to most oxysterols is related to their ability to induce apoptosis, through both the extrinsic and intrinsic pathways, and autophagy.

One oxysterol, 7-ketocholesterol (7KC), differs from free cholesterol by the addition of a functional ketone group at C7. 7KC has been extensively studied in different cellular models, where it has been reported to cause death through several mechanisms [9–12]. 7KC was shown to increase membrane permeability and reactive oxygen species (ROS) production, reduce the mitochondrial membrane potential, release cytochrome C, cause nuclear condensation and oxidative damage to DNA, and reduce nitric oxide production and cell proliferation [5, 8, 10–12].

Cell death promoted by oxysterols has been also observed in cancer cell models [13–15]. In view of the ability of 7KC to induce cell death, its pharmacological use in cancer therapy deserves to be addressed. Moreover, to avoid undesirable actions on normal cells, 7KC should rather be targeted to tumor cells. In fact, reducing the toxicity of different anti-cancer drugs is a major concern in cancer chemotherapy.

Cholesterol is an essential component for the stability and function of animal cell membranes [16]. Among the lipid molecules that form the membrane structure, roughly half are cholesterol. Malignant neoplastic cells require high levels of cholesterol for membrane synthesis, due to accelerated mitosis; thus, these cells upregulate their production of low density lipoprotein (LDL) receptors [17, 18]. This upregulation allows cancer cells to increase uptake of cholesterol-rich LDL particles. This observation led to the proposal that using LDL as a carrier could concentrate anti-cancer drugs in malignant cells and tissues. This hypothesis was successfully demonstrated in several different cancer types [19, 20]. However, the difficulties associated with incorporating drugs into LDL particles and isolating lipoproteins from human plasma have impeded the development of LDL-based chemotherapy for cancer patients. These difficulties were addressed with the development of nanoemulsions [21].

Practical use of the LDL receptor-mediated endocytic pathway was made possible by work from Maranhão *et al*. [22, 23]. They prepared artificial, lipid core nanoparticles with a composition that resembled LDL. When injected into the bloodstream, these nanoparticles could acquire apolipoprotein (apo) E, when they came into contact with native plasma lipoproteins. These apoE-carrying nanoparticles (LDE) could bind to LDL receptors through the apo E ligand, and thus, they were internalized into the cytoplasm. Furthermore, drugs associated with LDE could be concentrated inside cancer cells and tissues that expressed high LDL receptor levels, resulting in greater pharmacological activity and several-fold less drug toxicity [24–26].

Recently we have described a 7KC-containing nanoemulsion developed to provide an artificial nanoparticle suitable to study 7KC metabolism in lipoproteins [27]. This preparation (7KCLDE) was based on LDE composition and resembled LDL behavior. Here we tested the ability of 7KCLDE to cause cell death, to target tissues selectively and to decrease drug toxicity. The preliminary results from *in vitro* and *in vivo* experiments suggested that this novel preparation showed promising potential for future use in cancer chemotherapy.

## RESULTS

### *In vitro* studies

#### 7KCLDE uptake by LDL-receptor

**Figure 1:**
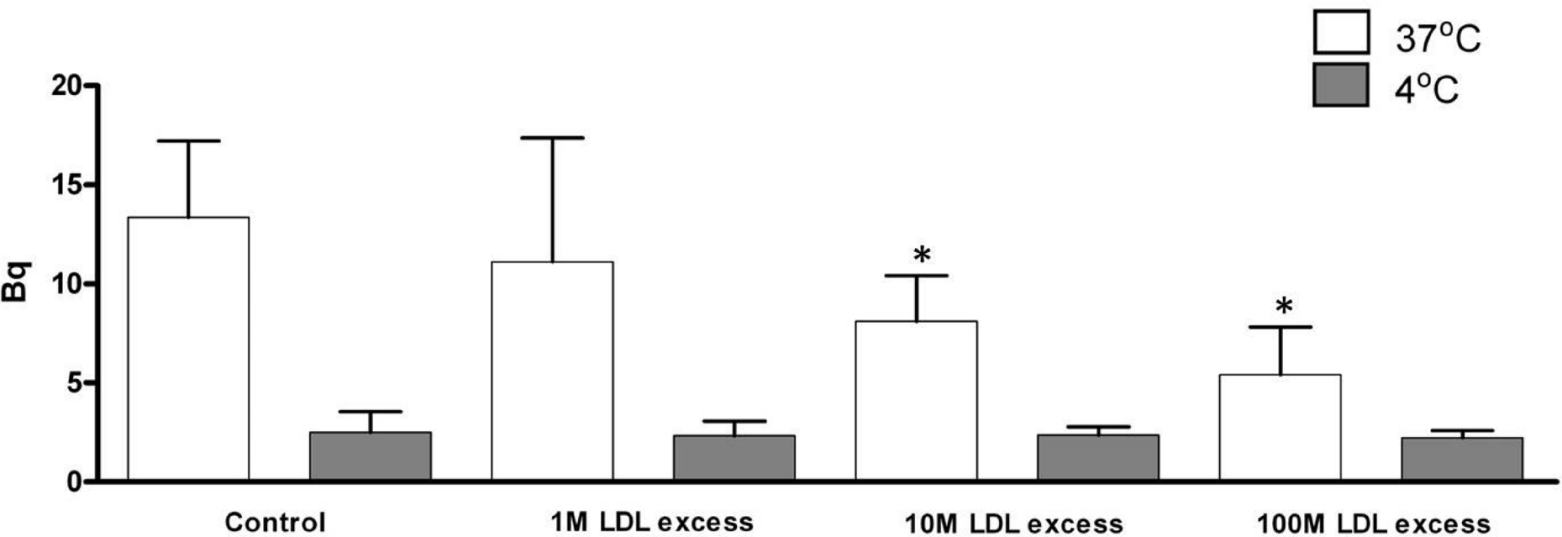
Uptake of 7KCLDE in the presence of native LDL B16F10 cells were incubated with 75 μM [^3^H]7KC/[^14^C]cholesterol-containing 7KCLDE and HDL (43 μg/mL), in either the absence or presence of LDL (1:1 up to 100:1 molar ratios of LDL:LDE) in serum-free medium for 4 h. The amount of radiolabeled material in cell lysates was determined with a LKB beta-counter. Each bar represents the mean ± SD of 6 independent experiments performed in triplicate.

Figure 1 shows the results of the competition experiments. There was a small uptake of 7KCLDE by melanoma cells incubated at 4° C, indicating that the receptor-independent pathway is less important. Uptake of 7KCLDE by cells incubated at 37° C was increasingly reduced by co-incubating with increasing amounts of native LDL. This finding strongly suggested that LDL and 7KCLDE were taken up by the same cell receptor mechanisms.

#### *In vitro* effects of 7KCLDE on B16F10 cell growth and death

**Figure 2:**
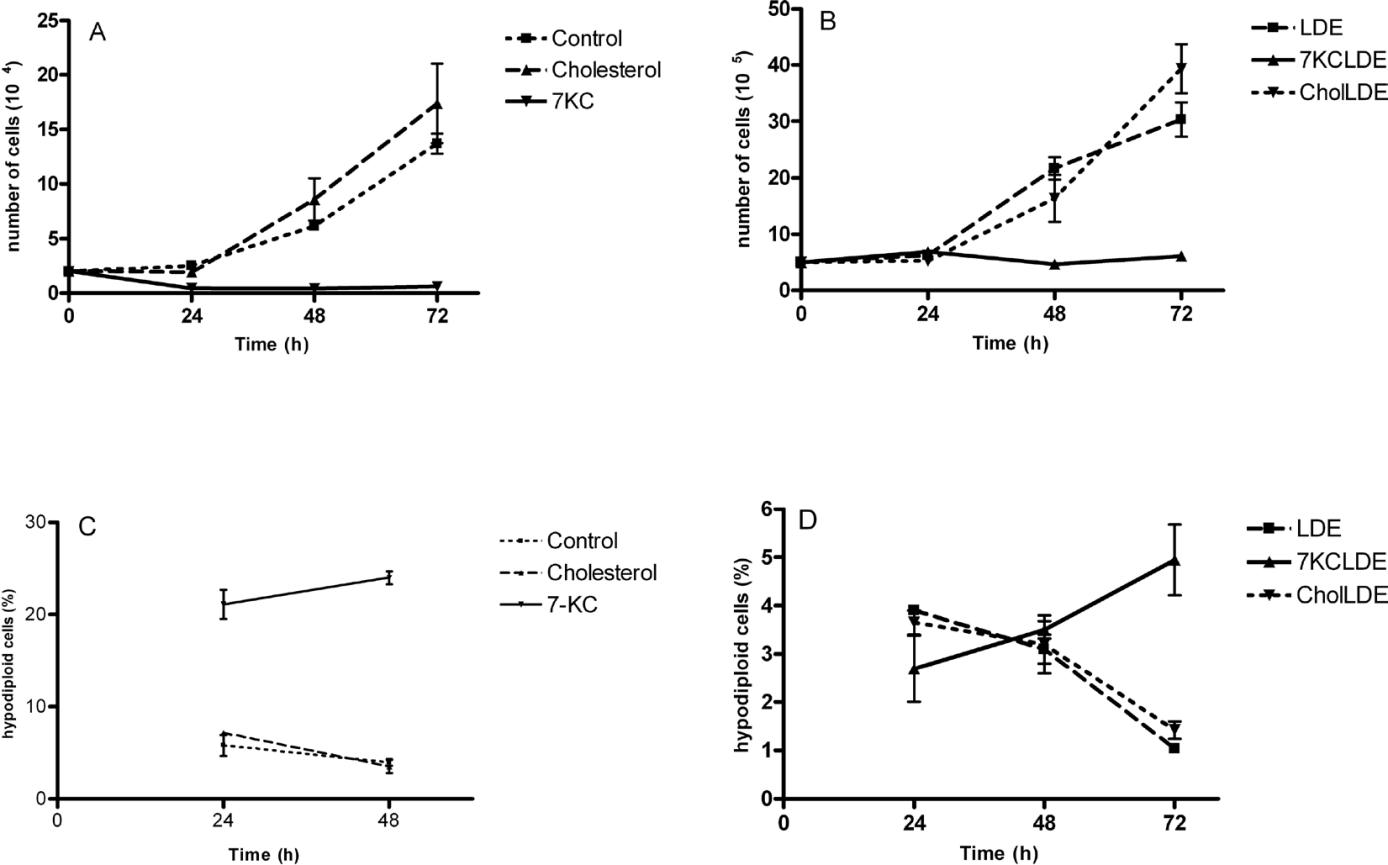
Cytotoxicity of 7KCLDE to B16F10 cells B16F10 cells were incubated with cholesterol (chol), LDE, CholLDE , or 7KCLDE for 1 to 3 days. (**A**, **B**) Cell viability was determined with trypan blue exclusion. (**C**, **D**) Cell cycle analyses were performed with flow cytometry; propidium iodide was used as a DNA-intercalating agent. Each point represents the mean ± SD of 6 independent assays performed in triplicate.

In an initial set of experiments, B16F10 cells were grown in the presence of 7KC or cholesterol, both diluted in ethanol, over a period of three days (Figure 2). Cells treated with 100 μM cholesterol showed the same doubling times as cells treated with ethanol alone (control) (Figure 2A). In contrast, cells treated with 100 μM 7KC showed growth arrest, and cell death (Figure 2A). Flow cytometric analysis of PI-stained cells treated with 7KC showed high proportions of hypodiploid cells (>20%) (Figure 2C). Next, melanoma cells treated with 7KCLDE were compared to two controls: LDE alone and LDE with an additional amount of cholesterol that corresponded to the concentration of 7KC (CholLDE). The two controls showed the same growth rates (Figure 2B). In contrast, cells treated with 7KCLDE showed growth arrest (Figure 2B) but, interestingly, no increase in the cell death rates were observed within the first 48 h, based on the proportions of hypodiploid cells (Figure 2D). After 48 h, cell death increased, but the rate was much lower than that observed with 7KC alone. Treatment with 7KC led to a decrease of cells in the proliferative phases of the cell cycle while treatment with 7KCLDE induced decrease of percentage of cells in G_0_/G_1_ (Supplementary Table 1). Thus, although a high concentration of 7KC induced cell death, as expected, 7KCLDE did not, at least at the same concentration.

**Figure 3:**
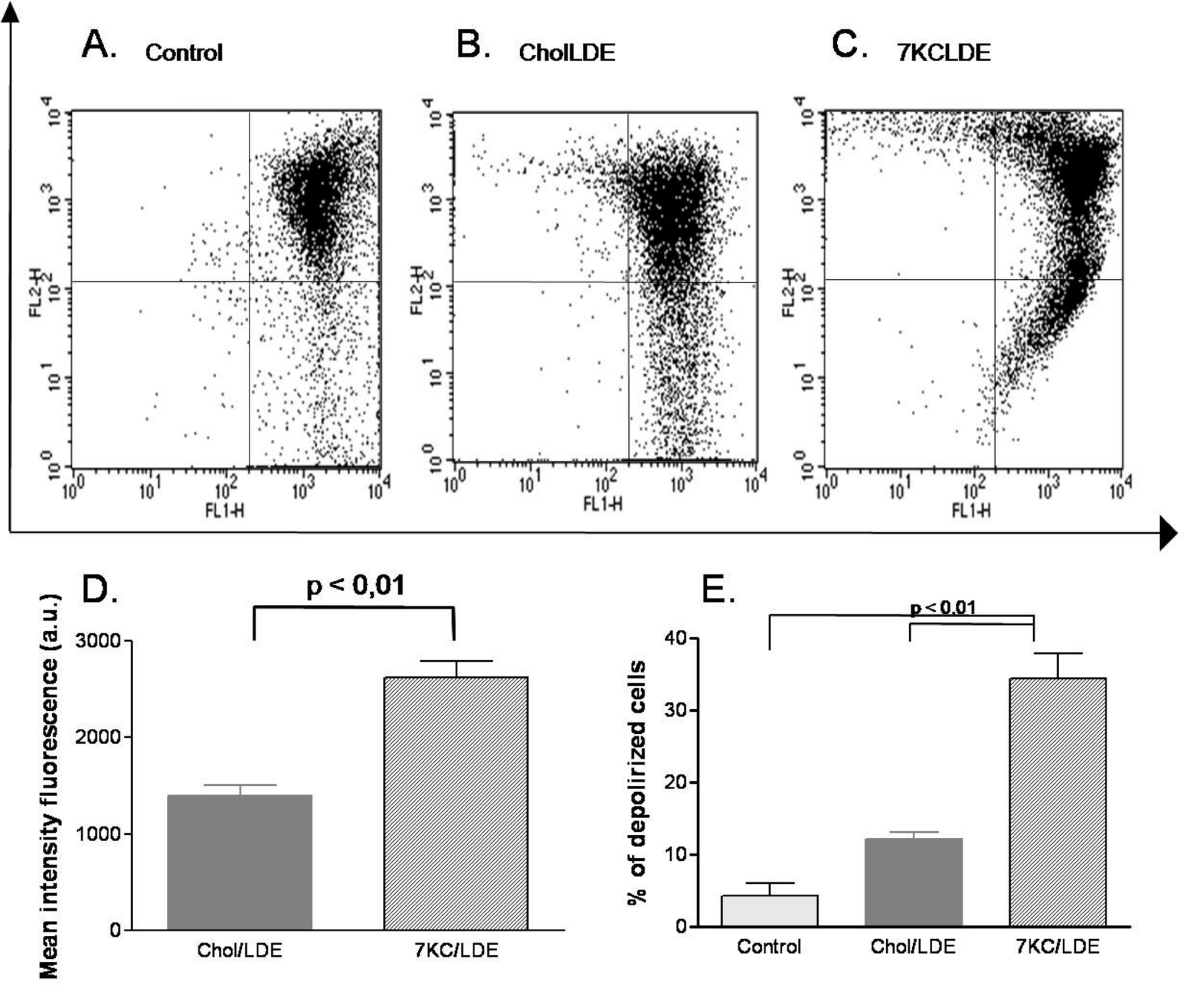
Effects of 7KCLDE on mitochondrial transmembrane potential (ΔY_m_) in B16F10 cells B16F10 cells were incubated with LDE or 7KCLDE for 24 h, and the ΔY_m_ was determined by staining with JC-1 (see Materials and Methods). Plots (**A–C**) represent the distribution of JC-1 aggregates as a function of JC-1 monomers. The ΔY_m_ is characterized by a reduction in JC-1 aggregates. (**D**) The mean fluorescence intensity of the upper right panel of each dot plot was quantified. The values represent the proportions of cells that harbored JC-1 concentrated in the mitochondria, which caused aggregate formation. Treatment of cells with 7KCLDE led to a significant increase in the mean fluorescence intensity of JC-1 aggregates. (**E**) Quantification of cells with dissipation of the mitochondrial transmembrane potential. The data indicate that 7KCLDE caused a loss of ΔY_m_ in around 30% of cells after a 24-h treatment. Bars represent the mean ± SD of 6 independent experiments performed in triplicate. ^*^p < 0.05; ^**^p < 0.01; a.u. = arbitrary unit.

Figure 3 shows that treatment with 7KCLDE for 24 h led to the dissipation of the mitochondrial transmembrane potential, measured as the loss of JC-1 aggregates. A significant increase in the fluorescence intensity of JC-1 aggregates was also observed in cells with an intact transmembrane potential (Figure 3D). This increase was previously ascribed to mitochondrial hyperpolarization [28], which precedes depolarization (Figure 3E). This phenomenon has been associated with mitochondrial release of cytochrome c, and it might elicit cell death via the apoptosis pathway.

**Figure 4:**
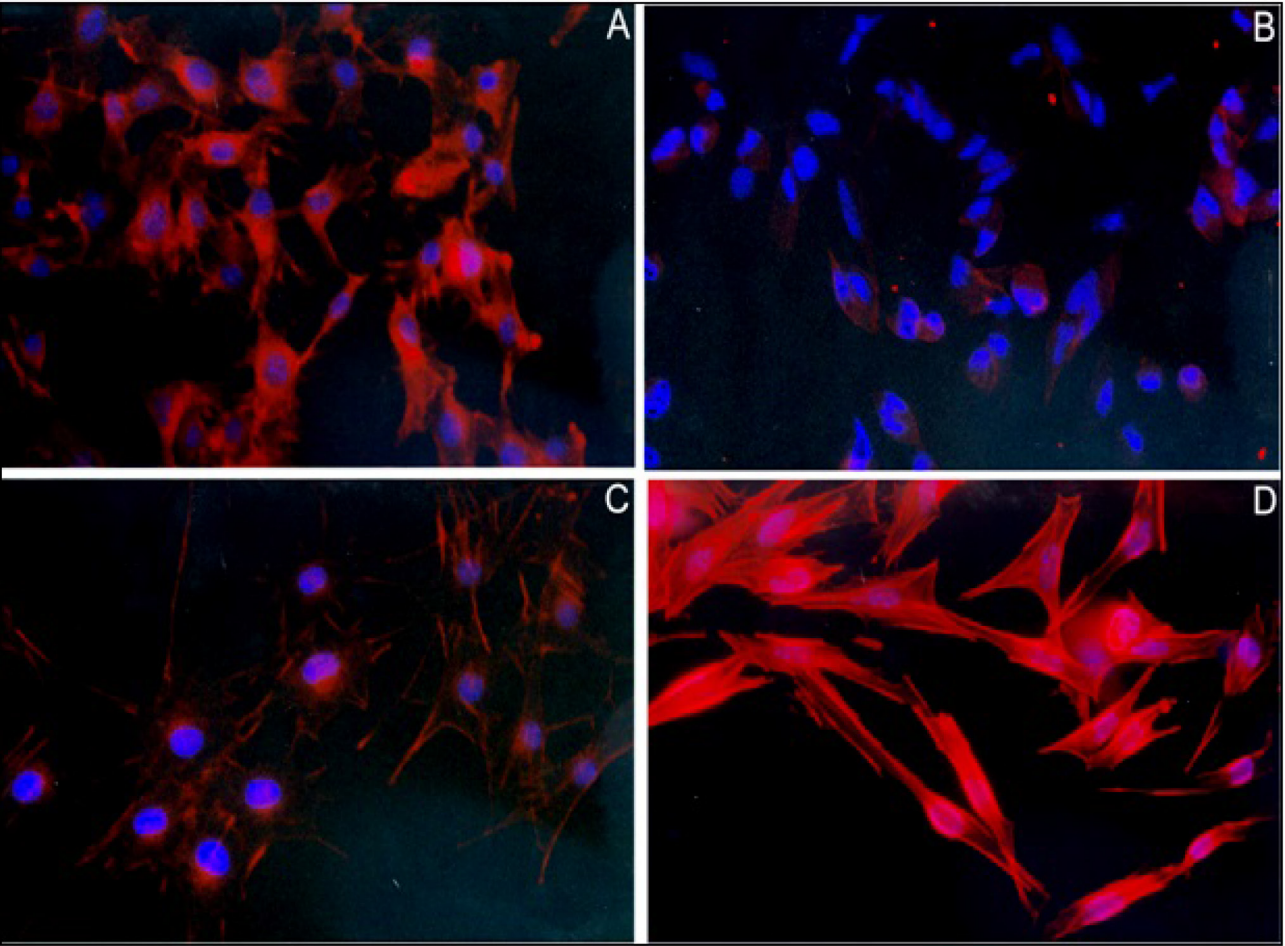
Effects of 7KCLDE on the formation of actin fibers in B16F10 melanoma cells B16F10 cells were cultured in the presence or absence of the indicated LDE emulsions for 24 h. (**A**) Under control conditions, melanoma cells were dendritic in shape. (**B**) Cells exposed to 7KC (75 μM) died, due to a loss of cytoplasm and detachment. (**C**) Cells incubated with LDE did not change in morphology. (**D**) Cells exposed to 7KCLDE (75 μM of 7KC) shifted in shape towards a fibroblastoid phenotype. This morphotypic change was associated with the formation of actin fibers, which was visualized with phalloidin-TRITC. Nuclei were visualized with DAPI. The nuclear shape changed with the phenotypic shift. Photomicrographs show representative, high power fields (magnification, ×400).

The morphology of surviving cells led us to further explore the action of 7KCLDE. Upon 24 h of exposure to 7KC, the cells lost cytoplasm and began to detach from the plastic dish (Figure 4B). After treatment with 7KCLDE (Figure 4D), but not LDE (data not shown) or CholLDE (Figure 4C), melanoma cells shifted from the epithelioid (Figure 4A) to a fibroblastoid (Figure 4D) morphotype. Spindle-shaped cells became increasingly abundant in cell cultures treated with 7KCLDE. This change in morphology was associated with increased polymerization of the actin cytoskeleton, evidenced with phalloidin staining (Figure 4D). Interestingly, the changes observed were not associated with the formation of typical stress fibers. Some binucleated cells were also found among 7KCLDE-treated melanoma cells. This finding prompted us to investigate whether cell growth arrest and altered cytoskeleton organization were associated with impaired cytokinesis.

**Figure 5:**
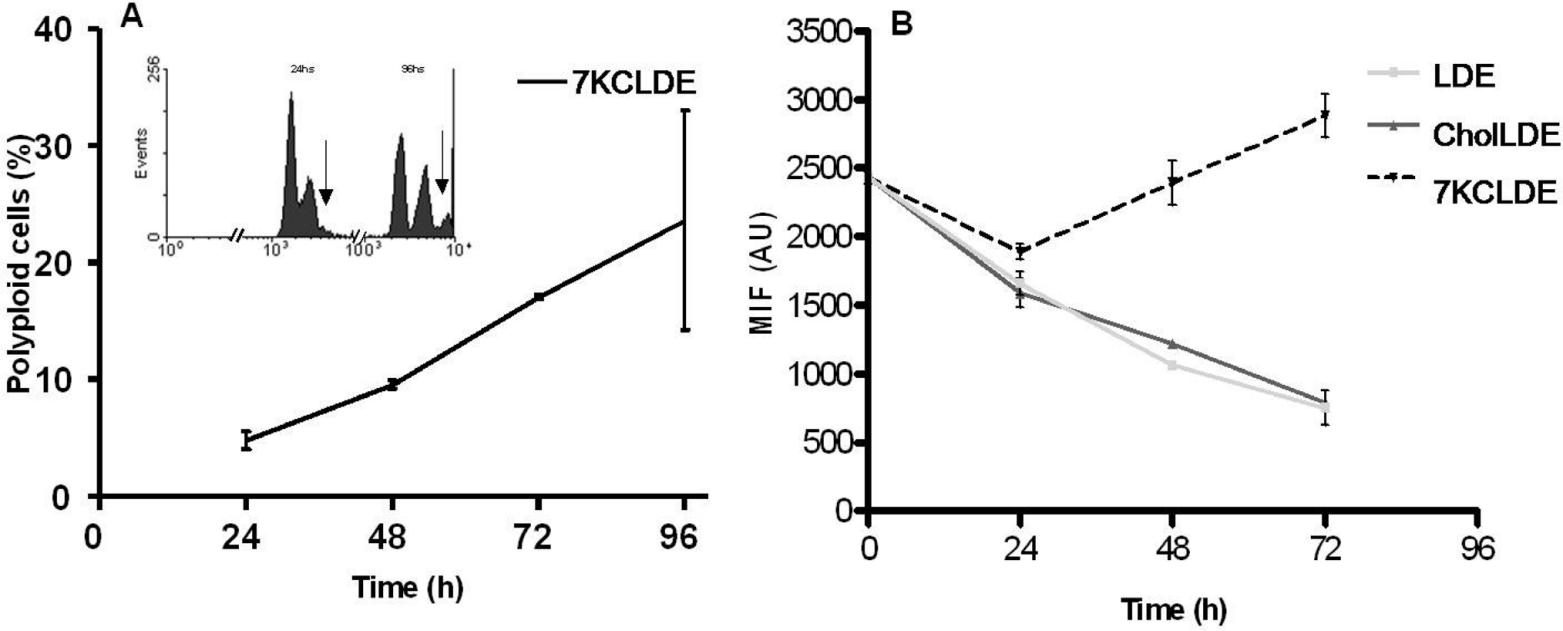
LDE7KC-induced polyploidy, associated with impaired cytokinesis B16F10 cells were incubated with LDE, CholLDE, or 7KCLDE for 1 to 4 days. Media containing the different emulsions were replenished every day. DNA content was analyzed by detecting propidium iodide with flow cytometry. (**A**) Emergence of a polyploid population (arrows) after 4 days of treatment with 7KCLDE. The fraction of polyploid cells increased progressively during treatment with 7KCLDE, as indicated. Points represent the mean ± SD of 5 independent assays performed in triplicate. No polyploid population was observed when cells were treated with either LDE or CholLDE (data not shown). (**B**) The separation of daughter cells after mitosis. Cells were pulse-labeled with the lipophilic dye, DiIC_18_, and chased for 1 to 3 days. Distribution of the fluorescent dye in post-mitotic cells was analyzed with flow cytometry. In both LDE-treated and CholLDE-treated cells, cell division was followed by a decline in the amount of fluorescent dye/cell. Cells treated with 7KCLDE did not lose fluorescent dye, suggesting that cytokinesis was halted. Each point represents the mean ± SD of 5 independent assays performed in triplicate. MIF: Mean Intensity Fluorescence.

Figure 5A shows that long exposure to 7KCLDE caused significant increases in the number of polyploid cells, and significant reductions in the proportions of cells in G_o_/G_1_ phase. The flow cytometry results suggested that the increase in polyploid cells might be due to DNA uptake; i.e., the surviving cells might phagocytose the dying cells, which would account for the elevated DNA content. Alternatively, polyploid cells could occur when cells replicated their DNA, but then, were arrested in mitosis. To investigate this mechanism, we pulse-labeled B16F10 cells with the lipophilic fluorophore, DiIC_18_, and chased it for three consecutive days in the experimental conditions shown in Figure 5B. As cells divided, the lipophylic dye was distributed randomly between the daughter cells. Thus, with normal cytokinesis, a progressive decline is observed in the mean fluorescence intensity per cell. Treating B16F10 cells with either LDE or CholLDE did not impair cytokinesis (Figure 5B). However, when cells were treated with 7KCLDE, the DiIC_18_-pulsed cells exhibited high fluorescence intensity over the entire assay period. This finding indicated that 7KCLDE strongly interfered with mitosis, and growth arrest resulted. Therefore, 7KCLDE has cytostatic action. The discrete, but significant, increase in the fluorescence intensity on day 3 (Figure 5B) suggested that 7KCLDE-treated cells might take up the dye released from dying cells.

**Figure 6:**
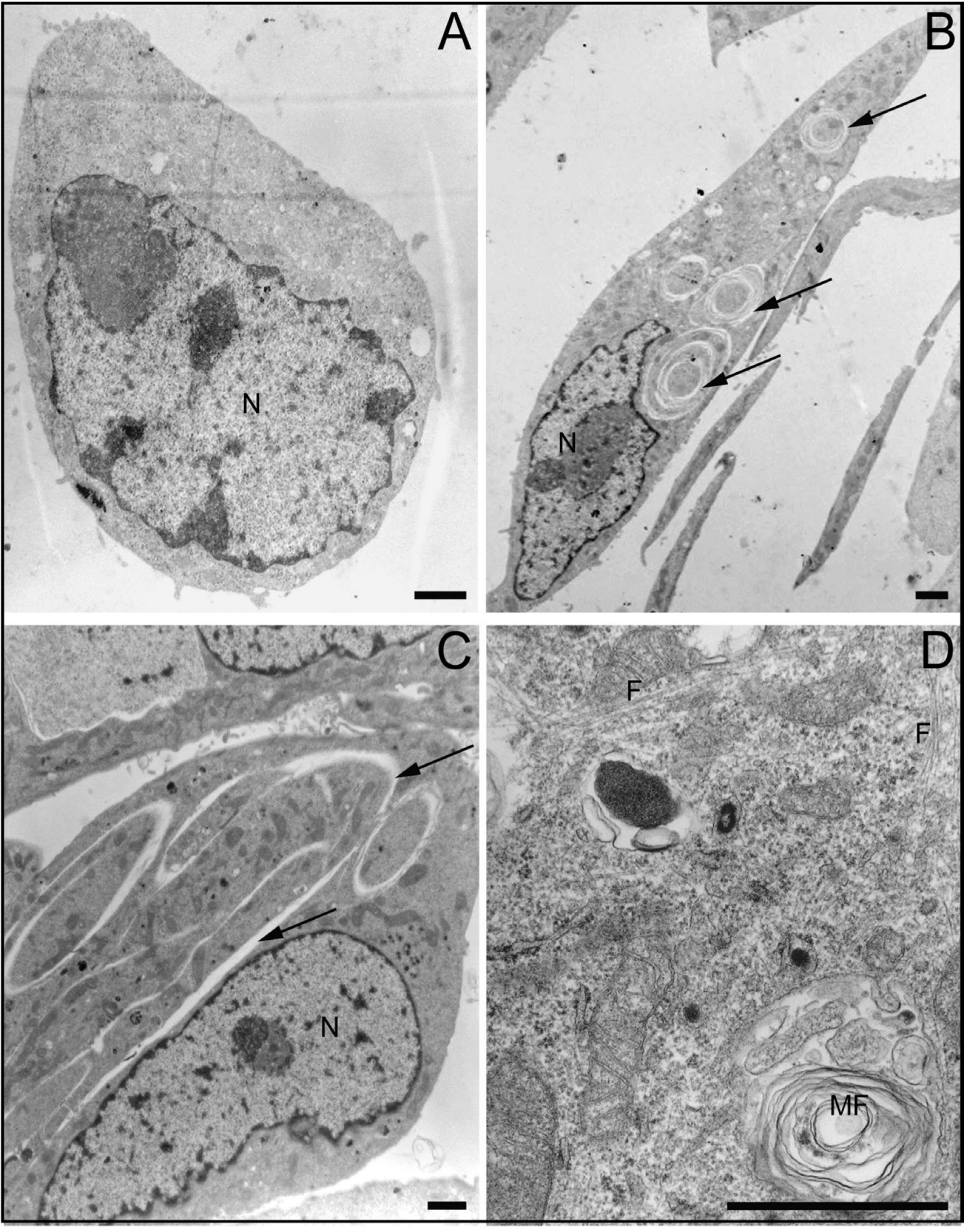
Ultrastructural changes in LDE7KC-treated cells Micrographs show (**A**) CholLDE-treated cell, and (**B–D**) 7KCLDE-treated cells. B16F10 melanoma cells shifted towards a fibroblastoid morphotype. (B, C) Nuclei (N) of 7KCLDE cells displayed morphological changes consistent with changes that led to the generation of structural ruptures in the cytoplasm (arrows). (D) Micrograph shows bundles of microfilaments (F) and autophagolysosomes (MF, myelin figures) observed after treatment with 7KCLDE. Scale bars indicate 1 μm.

**Figure 7:**
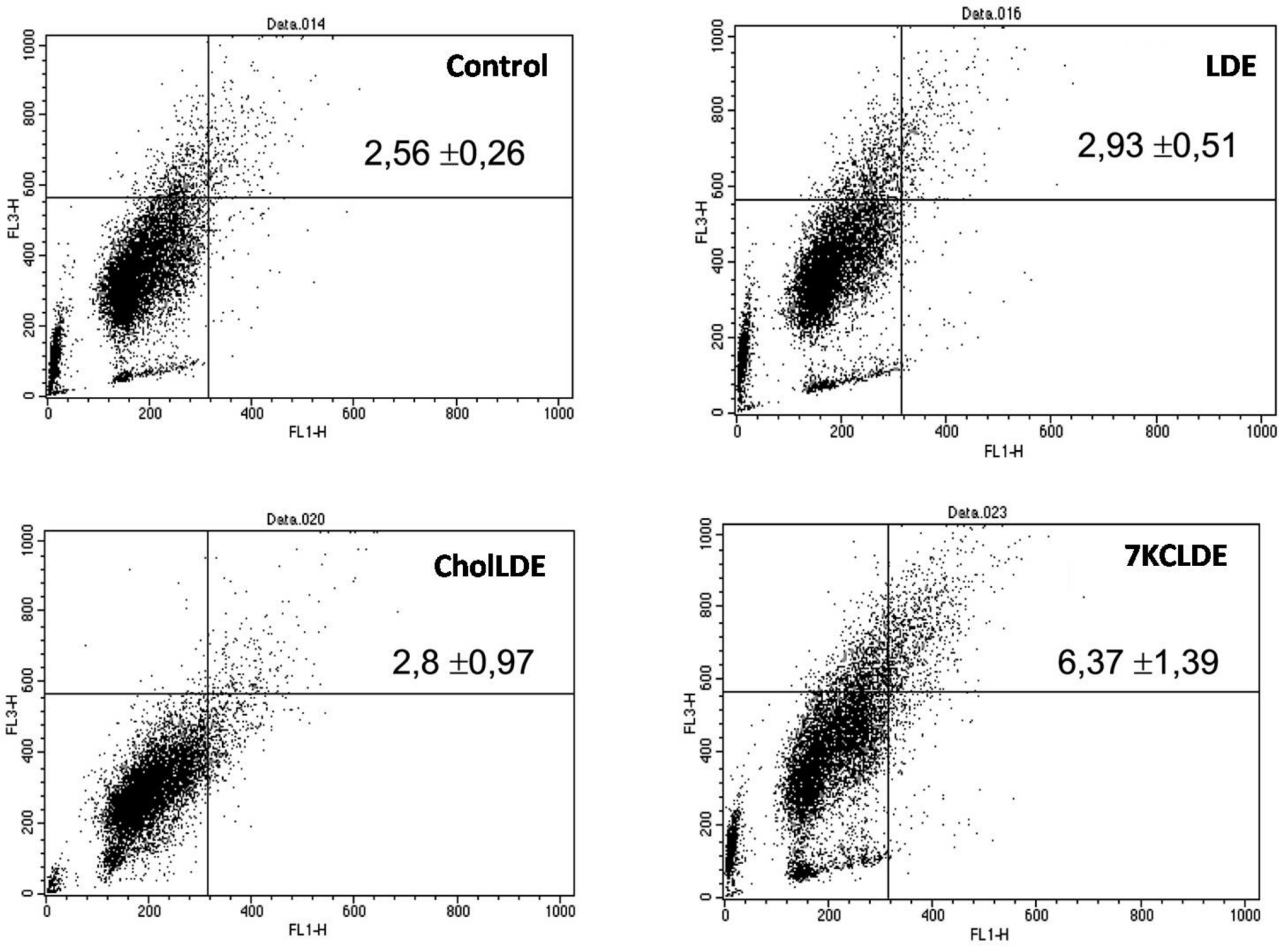
Acidic vacuole accumulation in B16F10 cells treated with 7KCLDE Melanoma cells were incubated with the indicated emulsions for 24 h and treated with acridine orange, which stains acidic compartments in bright red fluorescence. The upper right panels of flow cytometry dot plots represent cells with acidic compartments. Numbers indicate the relative frequency ± SD of a representative experiment performed in triplicate. 7KCLDE treatment increased (*p* < 0.05) cells harbored acidic vacuoles.

Ultrastructural analyses of cells treated with CholLDE (Figure 6A) and 7KCLDE (Figure 6B-D) allowed us to characterize the subcellular effects of 7KCLDE. No alterations were observed when B16F10 cells were treated with CholLDE (Figure 6A). However, 7KCLDE-treated B16F10 cells underwent a morphotypic conversion to a spindle-shaped cell (Figure 6B). We commonly found disruptions of the cytoplasm and formation of concentric structures (Figure 6B, 6C). Note that the nuclei changed shape, according to the impedance caused by these lamellar structures; this finding suggested that the structures were associated with a rigid material within the cytoplasm of 7KCLDE-treated cells. Bundles of microfilaments were found scattered in the cytoplasm (Figure 6D); this ultrastructural finding seemed to be related to the increase in actin fiber polymerization (Figure 4D). Finally, we found multilayered vacuoles that resembled autophagolysosomes in cells treated with 7KCLDE (Figure 6D).

Autophagolysosomes are acidic compartments that can be measured with flow cytometry, based on a property of the fluorophore, acridine orange. Acridine orange stains acidic compartments in organelles with bright orange fluorescence. In control cells, 2.56 ± 0.26% of cells harbored acidic compartments. However, with 7KCLDE treatment, 6.37 ± 1.39% (*p* < 0.05) of cells harbored acidic vacuoles (Figure 7). These results were confirmed with the fluorophore, monodansylcadaverine, which stains autophagic vacuoles (data not shown).

### *In vivo* studies

#### Plasma kinetics of 7KCLDE in healthy mice with melanoma implants

Following a single intravenous bolus injection of double-labeled 7KCLDE, both ^3^H-7KC and free ^14^C-cholesterol levels progressively declined in the plasma of control mice (Supplementary Figure 1A). A similar decay curve was observed in mice with melanoma implants (Supplementary Figure 1B).

#### Tumor uptake of 7KCLDE

In control animals, the 7KCLDE complex was taken up mainly by the liver, and, to a much lesser extent, by the adrenal glands, demonstrated by measurements of ^3^H-7KC and free ^14^C-cholesterol levels (data not shown). In animals with tumors (Supplementary Figure 2), liver uptake was higher than in controls. However, the tumors also took up 7KCLDE, and tumor content accumulated over time.

### Melanoma-bearing mice treated with 7KCLDE

Melanoma-bearing mice were treated daily with intraperitoneal injections of 7KCLDE, LDE alone, or saline (control). Hematological and biochemical analyses showed that 7KCLDE had no apparent toxicity. No toxicity was detected on hemograms, myelograms, or spleen cellularity analyses (data not shown), at the administered doses. This finding suggested that this nanoemulsion was well tolerated.

**Figure 8:**
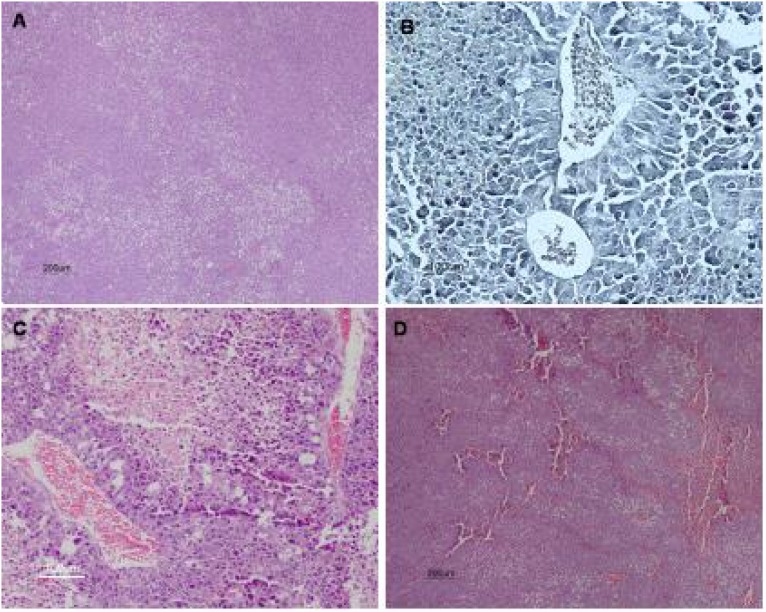
Histological analysis of melanoma tumors Ten days after injection of 5 × 10^4^ B16F10 cells rats were treated with 7KCLDE (156 mM, 100 μL volume IP) for other 10 days (*n* = 7). Animals were then sacrificed and tumors examined. Microscopic images show various tumor regions: (**A**) tumor (H&E staining, ×40); (**B**) blood vessels (Verhoeff staining, ×200); and (**C**, **D**) necrotic areas (H&E staining, C: ×100, D: ×40)

**Figure 9:**
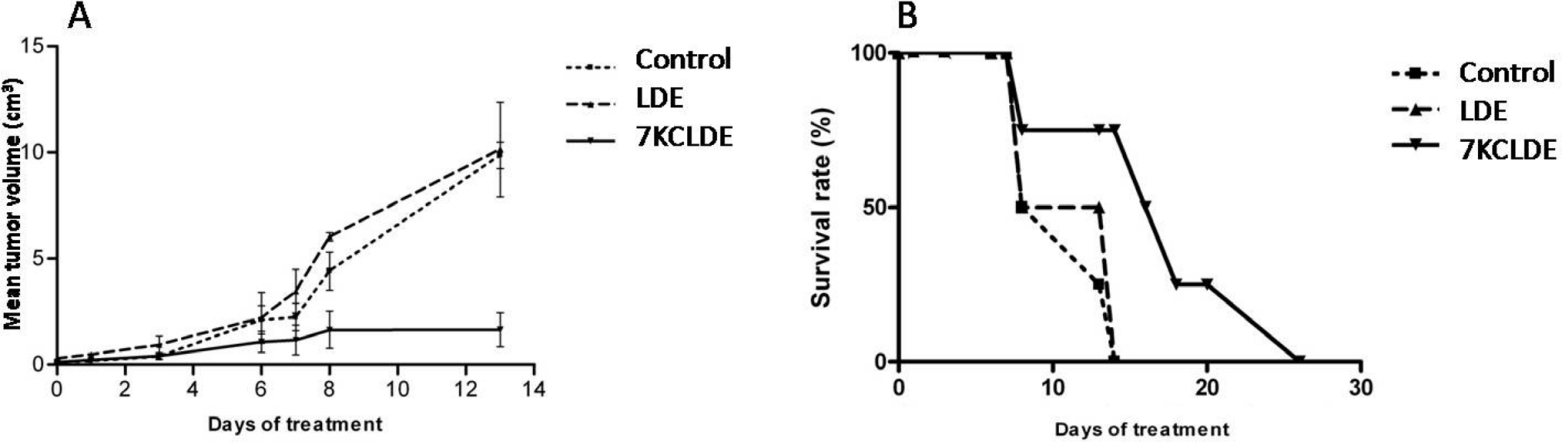
Effects of 7KCLDE *in vivo* (**A**) 7KCLDE reduced tumor volume. C57bl/6 mice were injected with B16F10 melanoma cells. After 10 days, mice were randomly divided into three groups to receive treatment with 7KCLDE (*n* = 8), LDE, (*n* = 7), or saline (control, *n* = 5) for 10 consecutive days (see material and methods). A significant reduction in tumor volume was observed in mice that received daily injections of 7KCLDE. Results are the mean ± SD; *p* < 0.05 (One Way ANOVA). (**B**) 7KCLDE treatment increased animal survival. Plot shows the percentage of B16 melanoma-bearing mice that survived in response to 7KCLDE treatment. Treatment prolonged survival by up to 15 days (*p* < 0.05; Kaplan-Meier Survival Analysis).

Figure 8 shows typical histological images of tumor tissues. The histological analysis of tumors showed that tumors from 7KCLDE-treated mice had larger necrotic areas (53.4 ± 7.8%) than tumors from control mice (18.0 ± 5.54%, *p* < 0.001) (Figure 8C, 8D). We also estimated the tumor vascular area with Verhoeff impregnation. The results showed that tumors from mice treated with 7KCLDE had smaller vascular areas (4.95 ± 4.27%) than tumors from control mice (9.8 ± 2.76%; Figure 8B). After 12 days of treatment, we observed a significant reduction in the tumor volume, promoted by 7KCLDE (Figure 9A).

7KCLDE treatment also affected mouse survival rates. Mice treated with 7KCLDE had a higher mean survival rate (Figure 9B) than mice treated with control (saline) or LDE solutions. Generally, animals treated with 7KCLDE lived up to 15 days longer than mice in other treatment groups.

## DISCUSSION

It is well known that bioactive lipids promote activation or regulation of several cellular processes [29–31], such as cell growth, proliferation, differentiation, and cell death [32, 33]. Among these compounds, oxysterols are potent, biologically active molecules. They are involved in a variety of functions, including the inhibition of cell proliferation and the promotion of cell death [34, 35]. Among all cholesterol oxides, 7KC has been extensively studied. When added to cell cultures, 7KC led to the formation of ROS, which induced lipid peroxidation and protein oxidation [36]. The protein modifications included both reversible and irreversible oxidation of sulfhydryl (−SH) groups found in proteins of essential cellular pathways, such as glutathione reductase, calcium ATPases, and actin [36]. These modifications were ultimately associated with cell death, either through classical apoptosis or by induction of autophagy. In fact, 7KC induced a particular mode of cell death, termed oxiapoptophagy, which simultaneously involved oxidation, apoptosis, and autophagy [8, 36]. The cytotoxic effects of 7KC have been demonstrated in both nontumorigenic and tumorigenic cell lines [11, 12, 37–41]. Here, we extended those observations to murine melanoma cells. Furthermore, we devised a strategy for delivering 7KC to cells in a controlled manner.

Several drug delivery systems that carry anti-cancer chemotherapeutic agents have been tested in patients with cancer, including liposomes, lipid core nanoparticles, albumin nanoparticles, and polymeric nanoparticles. Natural and synthetic HDL and LDL particles have also been explored for their use as vehicle for drug delivery [42–44]. Nevertheless, in a literature review of the past 10 years, it was reported that only 0.7% of administered nanoparticles was delivered to solid tumors [45]. LDE consists of a core of cholesteryl esters with residual triglycerides, surrounded by phosphatidylcholine and some unesterified cholesterol. LDE was probably the first non-liposomal drug delivery system that showed clear drug targeting properties [23]. Moreover, the toxicity of chemotherapeutic agents declined more intensely when associated with LDE [46, 47].

In this study, we intend to create an anti-cancer agent by adding an oxidized cholesterol derivative, 7-ketocholesterol, to LDE components. In melanoma cells, 7KC increased cell death, evidenced by the percentage of hypodiploid cells. However, upon emulsification with LDE, the percentage of hypodiploid cells was reduced. This could be explained, at least in part, by the fact that some deleterious effects of oxysterols *in vitro* can be diminished when they are combined with cholesterol or even with other oxysterols [48, 49]. 7KCLDE induced cell death by dissipating mitochondrial transmembrane potential and by causing DNA cleavage and acidic vacuole accumulation. Moreover, the acidic vacuoles were consistent with autophagolysosomes, based on the ultrastructural analysis (presence of myelin figures). Therefore, 7KCLDE induced pathways involved in both apoptosis and autophagy, consistent with descriptions of cells exposed to 7KC [11, 36, 50]. In our model, J-aggregates accumulated in the mitochondria of a large proportion of cells. This finding was previously described as indicative of mitochondrial hyperpolarization, which precedes dissipation of the transmembrane potential in apoptosis [28].

Therefore, 7KCLDE induced cell death in a significant fraction of melanoma cells, but it showed less potency than 7KC alone. The surviving cells invariably showed cytoskeletal rearrangements and were growth-arrested. The 7KCLDE cytostatic effect was not associated with arrest in the G_o_/G_1_ phases. In fact, DNA replication seemed to occur normally, based on the increasing frequency of polyploid cells. Disruption of cytoskeletal dynamics, as indicated by the increased polymerization of the actin cytoskeleton, may have impaired cytokinesis, which in turn, led to the emergence of a polyploid cell population. Increased actin polymerization was not related to the formation of stress fibers, suggesting that 7KCLDE induced an aggregation of actin fibers, rather than controlled polymerization. The presence of these aggregates within the cytoplasm was related to changes in nuclear morphology and the generation of concentric figures found within the cytoplasm at the ultrastructural level. The cytostatic effects of 7KCLDE were long-lasting, and they were not reversed when the emulsion was washed out.

The polymerization of actin is a laborious process that involves many proteins [51–53]. These proteins cleave, transport, and anchor actin to the plasma membrane. Some proteins act indirectly; for example, p38 MAP kinase modulates the reorganization and phosphorylation of heat shock protein 27, an important protein involved in oxidative stress-induced alterations in the cytoskeleton [28]. The cytoskeletal changes elicited by 7KC may be associated with the production of ROS [54, 55], which may activate MAP kinase and lead to actin polymerization. Moreover, it has been demonstrated that oxidative stress can disrupt the microfilaments of the cytoskeleton [55, 56].

When injected intraperitoneally into melanoma-bearing C57bl/6J mice, radioactive 7KCLDE was mainly found in the liver and in the tumor. Moreover, the tumor concentration increased over time. We found in these preliminary studies that 7KCLDE controlled tumor growth *in vivo*, which suggested that the *in vitro* effects of 7KCLDE may also operate *in vivo*. Furthermore, 7KCLDE did not cause any overt adverse reactions in treated mice. Our biochemical analyses included AST and ALT hepatic enzyme levels, and hematological analyses. These findings warrant more pharmacodynamic and preclinical studies of 7KCLDE.

Strikingly, the 7KCLDE-treated mice 50% survival was roughly the double (16 days) than untreated animals (8 days) and higher than LDE-treated animals (12 days).

In summary, this study showed that it was possible to create a 7KC-based bioactive nanoparticle with potential applications as an antiproliferative agent. Although much more pre-clinical studies are needed, including the testing of other cell lines and efficacy of different doses in terms of melanoma (and other tumors) progression and survival rate, the preliminary results strongly suggest that this novel 7KCLDE nanoparticle has promising potential for cancer therapy applications.

## MATERIALS AND METHODS

### Materials

Egg phosphatidylcholine, triolein, cholesteryl oleate, cholesterol, and 7KC were purchased from Sigma-Aldrich (St Louis, MO, USA). Lipids were 98% pure, determined with thin layer chromatography. [^14^C]-cholesterol (specific activity: 49 Ci/mmol) was acquired from Amersham (Little Chalfont, UK); [1,2,6 ^3^H]-7-ketocholesterol (5-cholesten-3β-ol-7-one, specific activity: 50 Ci/mmol) was acquired from American Radiolabeled Chemicals (St Louis, MO, USA), 99% pure. Cell culture materials were purchased from Invitrogen (Carlsbad, CA, USA). All other materials were from Sigma-Aldrich.

### Ethics statement

The investigation has been conducted in accordance with ethical standards and national and international guidelines. The protocol of the study was approved by the Ethics Committee of the University of São Paulo Medical School (CAPEPesq 391/10).

### Preparation of 7KC-containing nanoparticles

7KCLDE was prepared as described [27] based on the method for LDE preparation [22, 23]. Briefly, a lipid mixture (5.0 mg 7KC, 40 mg cholesteryl oleate, 20 mg egg phosphatidylcholine, 1.0 mg triolein, and 0.5 mg cholesterol in aqueous medium) was exposed to ultrasonic irradiation. Previous results showed that 5.0 mg 7KC would be most appropriate for the experiments [27]. [^3^H]-7KC was added to each nanoemulsion preparation to enable evaluation of the amount of incorporated 7KC. When necessary, radioactively labeled lipids ([^3^H]-7KC and [^14C^]-cholesterol, 9 × 10^6^ cpm each) were added to the lipid mixtures, as indicated in the experimental protocols. Next, a two-step ultracentrifugation procedure was performed. The resulting 7KCLDE samples were dialyzed against a saline solution and sterilized by passing through a 0.22 μm filter. Control preparations were made without 7KC (LDE) or by replacing 7KC for 5.0 mg free cholesterol to the lipid mixture (CholLDE). The particle sizes in the different preparations were determined with laser light scattering (Zeta Potential Analyzer, Brookhaven Instruments, Holtsville, NY). Preparations were used only when the nanoparticle diameters were in the range of 20-50 nm, the same measured by the LDE alone and by native LDL. The nanoparticles were stable for at least one month, evidenced by visual inspection and by measuring the diameters with laser light scattering. Nevertheless, they were used within one week after preparation.

### Cell culture

B16F10 melanoma cells were grown in RPMI 1640 medium supplemented with 10% fetal bovine serum (FBS) at 37° C in a humidified atmosphere containing 5% CO_2_ [57]. After thawing [58], cells were seeded at an initial density of 2 × 10^4^ cells/cm^2^. Upon 80–90% confluence, cells were harvested with trypsin/EDTA. Culture contamination by mycoplasma was routinely tested by PCR. Cell viability was determined by Trypan Blue exclusion (Invitrogen, Carlsbad, CA), and by direct counting in a hemocytometer.

### Competition assay between human plasma LDL and 7KCLDE

To clarify whether 7KCLDE internalization depended on LDL receptors, we measured uptake of radiolabeled 7KCLDE in the presence of increasing concentrations of human plasma LDL. HDL was added at a constant concentration, as an apoE donor. These assays were performed at 4° C and 37° C, as previously described [59]. Briefly, 2 × 10^4^ cells were seeded into a 24-well plate and incubated with 75 μM [^3^H]-7KC/ [^14^C]-cholesterol-containing 7KCLDE and 43 μg/mL HDL, either in the absence or presence of different molar ratios of LDL/7KCLDE (1:1, 10:1, 100:1) in serum-free medium for 4 h. Cells were then washed, harvested, and lysed. The amount of radiolabeled material in the cell lysates was determined in a LKB beta counter (Wallac, Turku, Finland).

### Cell cycle analysis

Cell cycle analysis was determined with flow cytometry. After the indicated treatments, 2 × 10^6^ cells were trypsinized, washed three times with PBS, fixed in 70% ethanol, and stained with propidium iodide (PI), as described [60]. All analyses were performed with a FACScalibur flow cytometer (Becton Dickinson, San Jose, CA). The red fluorescence signal from PI was detected through a 585/42-nm band–pass filter and measured on a linear scale of 1024 channels. For each sample, at least 10,000 events were acquired with CELLQuest software.

### Mitochondrial transmembrane potential

To analyze mitochondrial transmembrane potential (ΔΨM) variations, we used carbocyanine dye JC-1, which accumulates in the mitochondrial matrix and forms J-aggregates. After the indicated treatments, cells were trypsinized and washed with PBS. Then, 10^6^ cells were incubated in medium with 200 nM JC-1 at room temperature in the dark, in a humidified chamber for 15 min. Next, the cells were analyzed with flow cytometry. The emission at low membrane potential (green) was collected at 530 nm and the emission at high membrane potential (red) was collected at 585 nm [61]. All experiments were performed in triplicate. For each replica, data from 10,000 cells were acquired and analyzed with CELLQuest software.

### Supravital staining with DiIC_18_

Cells were labeled with DiIC_18_, a supravital fluorochrome that can be used as a marker of cell division. Cells were seeded into 6-well plates (2 × 10^6^ cells/well) and DiIC_18_ was added in a 30-min pulse. Then, cells were washed with PBS. Both control and 7KCLDE-treated cells were then analyzed in sets of triplicates with flow cytometry, daily for three days. As cells divided, the amount of DiIC_18_ in each cell decreased geometrically; thus, the mean fluorescence intensity was correlated with the number of cell divisions.

### Morphological analysis

Cells were seeded (5 × 10^4^) onto coverlips and cultured in the presence of 7KCLDE. Cells were then fixed with 1% buffered paraformaldehyde for 5 min at room temperature, washed three times with a solution of 2% bovine serum albumin (BSA) in PBS, and finally, incubated with TRITC-labeled phalloidin (1:400 v/v in PBS), for 30 min at room temperature in the dark. The nuclei were stained with 4ʹ,6-diamidino-2-phenylindole (DAPI) at 1:1000 v/v in PBS, for 5 min in the dark. Images were acquired with a digital camera connected to a Nikon Eclipse E600 fluorescence microscope.

### Electron microscopy

After the indicated treatments, 2.0 × 10^7^ cells were fixed in a 2% glutaraldehyde solution for 30 min, post-fixed in 2% osmium tetroxide for 10 min, dehydrated with acetone, and embedded in Epon. Thin sections were cut and stained with uranyl acetate and lead citrate. The sections were examined with a JEOL 1010 electron microscope.

### Analysis of intracellular acidic compartments

To analyze and quantify the presence of autophagic vacuoles, we added an autofluorescent compound, monodansylcadaverine (MDC, Sigma, St. Louis MO), which is a selective marker for autophagic vacuoles [62]. Cells (5 × 10^4^) were seeded onto coverslips, incubated with 7KCLDE, and then, incubated with 0.05 mM MDC in PBS at 37° C for 15 min. Cells were then washed 3 times with PBS and immediately analyzed under a fluorescence microscope. Different set of cells were also treated with different nanoemulsions for 24 h, and then were stained for 20 min at 37° C with acridine orange (1 μg/mL), which emits a bright red fluorescent light when concentrated within an acidic compartment. Stained cells were harvested with trypsin, resuspended in PBS, and analyzed with flow cytometry, with excitation at 488 nm with an argon laser.

### *In vivo* studies

Melanoma xenografts were created in C57BL/6J mice by injecting subcutaneously 5 × 10^4^ B16F10 melanoma cells into the left flank. Experiments were performed after tumors became palpable, 8–10 days after the injection.

### Plasma kinetics and biodistribution of double-labeled 7KCLDE

7KCLDE (0.3 mg) was labeled with ^3^H-7KC and free ^14^C-cholesterol. This double-labeled 7KCLDE was injected into mice as a single bolus delivered into the retro-orbital venous plexus (control, *n* = 7; tumor, *n* = 7). Blood samples were collected after 5 to 240 min. Prior to radioactivity counting, plasma was separated from the blood samples with 3000 × g centrifugation for 15 min.

After 4 h, animals were killed by cervical dislocation, and samples from several organs were collected and maintained in saline solution prior to weighing. Lipid extraction was performed with chloroform: methanol (2:1, v/v). Radioactivity was measured in a liquid scintillation solution.

### Tumor volume and rate of survival

We tried to determine both, the maximum tolerated dose and LD50, with a single intraperitoneal injection of 7KCLDE at varying concentrations, from 2.5 mg/kg (78.125 mM) to 20 mg/kg (625 mM). Volumes ranged from 100 μL to 2 mL. Animals were observed up to 30 days after the injection. No death or other adverse manifestations were observed in this period. Thus, based on the *in vitro* findings and the ease of handling, the concentration of 5 mg/kg (156 mM) in a volume of 100 μL was used in the experiments.

Once tumors became palpable, animals were given daily intraperitoneal injections of 7KCLDE (156 mM, 100 μL volume) for 10 days (*n* = 8). Controls received injections of the same volume of either LDE alone (*n* = 7) or saline (*n* = 5).

Plasma γ-globulin, transaminases, alkaline protease, total bilirubin, urea, and creatinine were determined at baseline and after ten days of treatment. Blood was collected from the axillary plexus. At the end of the experimental period, bone marrow was collected from a femur of each animal. Spleen cellularity was also analyzed.

Tumor size was measured with a caliper, and the volume was estimated with the formula V = π/6× (perpendicular diameter)^2^ × largest diameter [63]. Microscopical images were obtained after staining with H&E (necrotic areas) and Verhoeff (vascularization). Survival time was also recorded for each animal.

### Statistical analysis

All data are expressed as the mean ± SD for at least three independent experiments. Non-parametrics statistical tests (Kruskal–Wallis and Mann–Whitney *U* tests) were used to analyze the significance of the differences of blood biochemical parameters. Other data were analyzed by the two-tailed Student's *t* test for paired samples. One way ANOVA on Graph Pad Prism with the log-rank test was used for the survival data analysis. The level of statistical significance was set at *p* < 0.05.

## SUPPLEMENTARY MATERIALS FIGURES AND TABLES


