# Supplementary material for: Cell internalization of 7-ketocholesterol-containing nanoemulsion through LDL receptor reduces melanoma growth in vitro and in vivo: a preliminary report
Source: Oncotarget. 2018 Feb 4;9(18):14160–74. doi: 10.18632/oncotarget.24389 (PMC5865661; doi:10.18632/oncotarget.24389)
Supplement: Supplementary file 1 [file oncotarget-09-14160-s001.pdf]

Cell internalization of 7-ketocholesterol-containing nanoemulsion through LDL receptor reduces melanoma growth *in vitro* and *in vivo*: a preliminary report

SUPPLEMENTARY MATERIALS

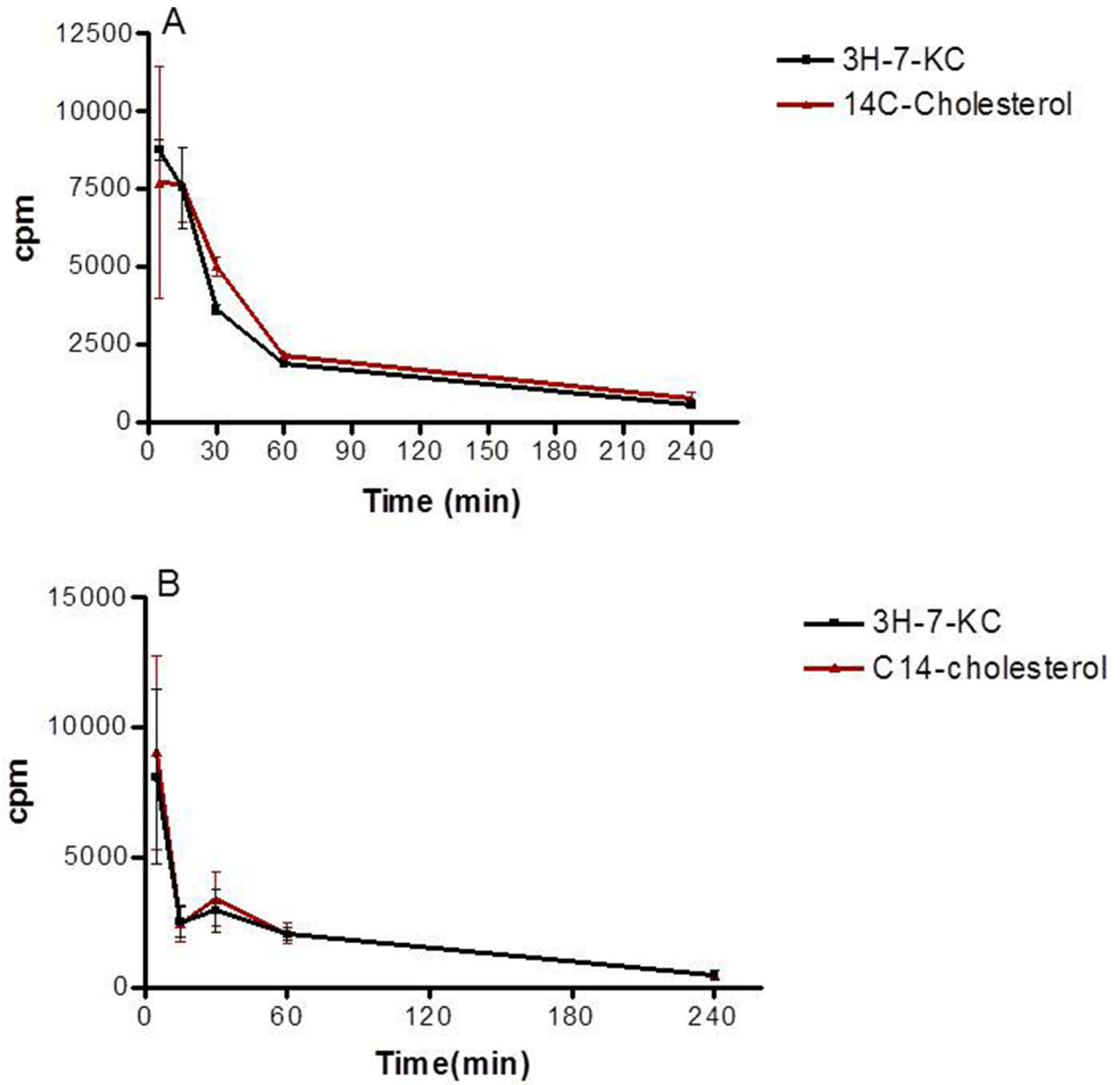

Supplementary Figure 1: Plasma decay curve of double-labeled 7KCLDE in mice. Curves show the disappearance of plasma 7KCLDE double-labeled (^{3}H-7KC and ^{14}C-free cholesterol), after a single bolus was injected into the retro-orbital venous plexus of mice. (**A**) Plasma 7KCLDE decay in healthy mice (n = 7). (**B**) Plasma 7KCLDE decay, when the bolus was injected 10 days after B16F10 melanoma cells were engrafted in mice (n = 7).

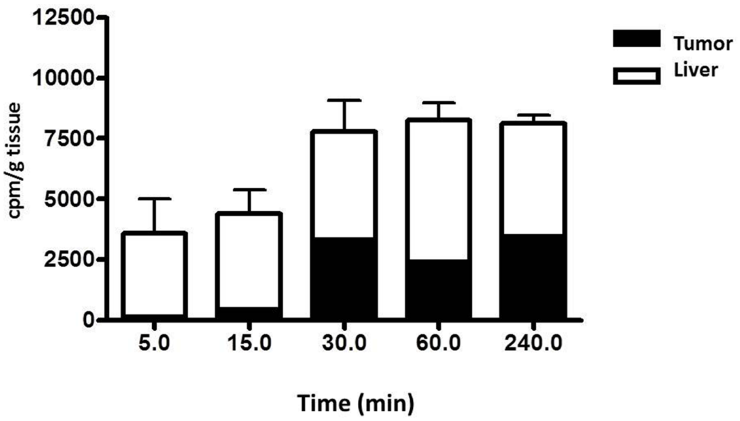

Supplementary Figure 2: 7KCLDE accumulation in melanoma tumors. Mice were injected with B16F10 melanoma cells. After 10 days, 7KCLDE was intravenously injected, and radioactivity was counted in different organs. The highest levels of radioactivity were found in the liver and the tumor. The level of radioactivity in liver was not different from that detected in controls. The small amount of radioactivity detected in other organs was not different from that detected in controls (data not shown). Results are expressed as the mean ± SD for 7 different animals.

Supplementary Table 1: Cell cycle evaluation by PI on B16F10 cells after treatment with cholesterol (Chol) or 7-ketocholesterol (7KC) alone or incorporated into nanoemulsion

	**24 h**	**24 h**	**48 h**	**48 h**
	**G_0/G_1(%)**	**$S/G_2/M$(%)**	**G_0/G_1(%)**	**$S/G_2/M$(%)**
Control	54.9 + 2.6	36.7 + 2.8	48.7 + 1.3	41.3 + 1.8
Cholesterol	57.9 + 1.9	32.6 + 2.6	52.2 + 2.0	45.3 + 3.5
7KC	54.7 + 1.7	25.0 + 5.6*	46.6 + 2.2	20.1 + 6.1*
CholLDE	47.0 + 7.0	39.2 + 11.9	44.2+ 5.6	38.1 + 9.4
7KCLDE	42.4 + 4.1*	33.6 + 9.4	40.3 + 6.7*	38.9 + 10.1

*$P < 0.05$ compared with controls.
